# Supplementary figures and images for: Small-Scale Randomized Controlled Trial to Explore the Impact of β-Hydroxy-β-Methylbutyrate Plus Vitamin D3 on Skeletal Muscle Health in Middle Aged Women
Source: Nutrients. 2022 Nov 4;14(21):4674. doi: 10.3390/nu14214674 (PMC9658601; doi:10.3390/nu14214674)

Supplemental Figure S1. Study Flow Chart.

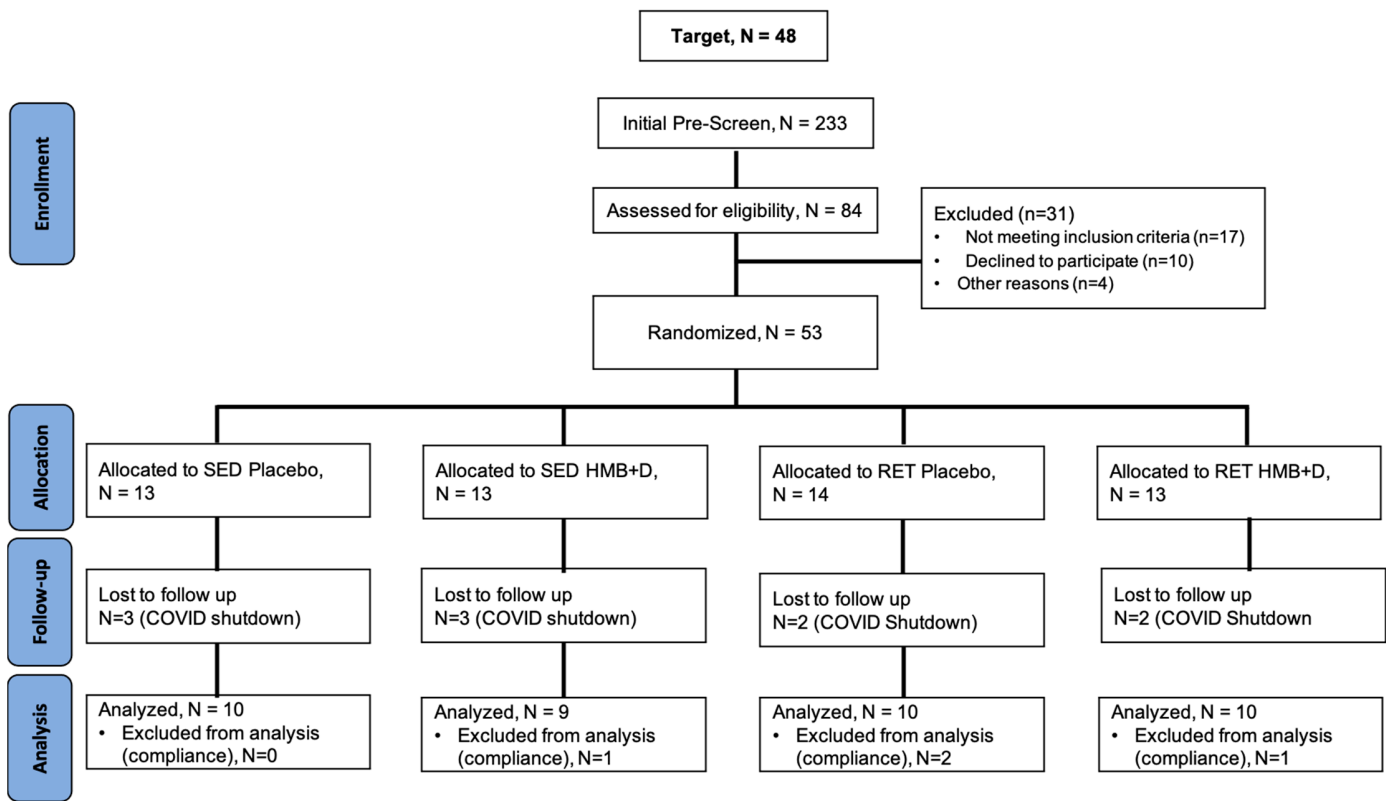

Supplement: Supplementary file 1 [file nutrients-14-04674-s001.zip › Supplemental Figure S1.pdf]
